# Evaluation of the Nutritional Quality of Breakfast Cereals Sold on the Italian Market: The Food Labelling of Italian Products (FLIP) Study

**DOI:** 10.3390/nu11112827

**Published:** 2019-11-19

**Authors:** Donato Angelino, Alice Rosi, Margherita Dall’Asta, Nicoletta Pellegrini, Daniela Martini

**Affiliations:** 1 Human Nutrition Unit, Department of Veterinary Science, University of Parma, 43125 Parma, Italy; 2 Human Nutrition Unit, Department of Food and Drug, University of Parma, 43125 Parma, Italy

**Keywords:** breakfast cereals, food labelling, nutrition declaration, nutritional quality, gluten free, nutrition and health claims

## Abstract

Breakfast cereals are present on the market as different types and, in general, are one of the food categories in which voluntary information, such as nutrition or health claims (NHC) or gluten free (GF) declarations, have the largest distribution. The aims of the present study were to compare (i) the nutritional declaration among different types of breakfast cereals, as well as among products with and without NHC or GF declarations; and (ii) the salt and sugar contents with the “Italian shared objectives for the improvement of the nutritional characteristics of food”. To this aim, the nutrition declarations of 371 different breakfast cereal items, available in 13 retailers present on the Italian market, were analysed. Data showed an elevated inter-product variability, with cereal bars and muesli having the highest energy, total fat, and saturate contents per 100 g. Limited differences were found comparing products with and without NHC, as well as those with GF declaration. Most of the breakfast cereals were compliant to the shared objectives, although some items with NHC or GF declaration still have sugar or salt contents higher than these objectives. In conclusion, these data suggest that the different characteristics and the regulated information reported on the food label should not be considered as a marker of the overall nutritional quality. Thus, this study supports the importance of reading and understanding the information made on food label.

## 1. Introduction

Breakfast is one of the most important meals of the day, as it comes after several hours of night fasting, and it literally “breaks the fast”. Several epidemiological and intervention studies evidenced a pivotal role of breakfast consumption in the maintenance of cardiovascular health [1,2], improvement of cognitive functions [3], and positive influence on satiety-related hormones [4]. Despite this important role, there is no agreement in the scientific community on its definition because there is no standard breakfast meal due to different cultures, food choices, and behaviours [5]. However, several studies agree that certain criteria should be followed in order to have an appropriate breakfast [5]. For the Italian population, it has been proposed that a balanced breakfast should provide 15% to 25% of daily energy for adults [2]. At least three food groups should be considered: milk and milk-derived products (low-fat), fruit (fresh fruit or 100% fruit juices), and cereals (preferably whole-grain, unrefined) [2,6]. Cereals have been endorsed as the principal source of breakfast’s carbohydrates [2] and allow the consumers to vary their breakfast meal with several different cereal-based products. Among these, breakfast cereals are nowadays available in numerous formulations and have been associated with the reduction of the risk of several chronic diseases in both adults and adolescents [7]. However, in Italy, breakfast cereals are still scarcely consumed—the total population has a median estimated intake close to 2 g/day, whereas adult habitual consumers (around 10% of the total population) have a median estimated intake of 15 g/day [8]. By comparing the intakes found in the European Prospective Investigation Cohort, the Italian intake is similar to those of other Mediterranean countries, such as Spain and Greece, and at least 20-fold lower compared to Scandinavian countries [9]. Other than a good source of available carbohydrates, breakfast cereals can be an important source of micronutrients (e.g., vitamins and minerals) and fibre, such as β-glucans, which play a key role in the prevention of cardiovascular risk, but also in the improvement in appetite control and increase of satiety [10,11]. However, breakfast cereals can also contain high amounts of added sugar and salt [12,13], ascertained risk factors of many chronic diseases when excessively ingested. For these reasons, there is a growing interest in the reformulation of breakfast cereal products. In Italy, as shown by the “Shared objectives for the improvement of the nutritional characteristics of food products” drafted by the Ministry of Health in collaboration with certain sectors of the food industry, the main reformulations for breakfast cereals were aimed to reduce sugar and salt up to mean contents of 30 g and 1 g per 100 g, respectively, by 2017 [14]. 

The first tool for the delivering of nutrition and health information to consumers is the food label. In Europe, mandatory and voluntary information made on food is regulated by specific laws including (i) the European Regulation (EU) no. 1169/2011, which regulates the mandatory information on foods, such as the list of ingredients and the nutritional declaration [15]; (ii) the European Regulation (CE) no. 1924/2006, concerning the voluntary Nutrition and Health Claims (NHC) [16]; and (iii) the European Implementing Regulation (EU) no. 828/2014, which regulates the information given to consumers on the absence or reduced presence of gluten in food [17]. Due to their nutritional composition, breakfast cereal is one of the food categories in which voluntary information, such as NHC, have the largest penetration [18,19]. However, on the basis of the so-called “health halo effect”, consumers might be biased and lead to generalizing the healthiness of these foods simply from some information present on the labels, such as NHC, regardless of the whole nutritional quality of the product [20,21]. Thus, there is a great interest in understanding if this information made on food can be considered as a marker of the overall quality of breakfast cereals.

With these presumptions, the aims of the present work were (i) to investigate the nutritional quality of breakfast cereals by collecting their nutritional values as declared on the food labels; (ii) to compare the energy and nutrient content of the products, classified for different characteristics (type of product, presence/absence of nutrition or health claims, gluten free (GF) declaration); and (iii) to compare the salt and sugar contents of all the products with the mean contents expected in breakfast cereals in the “Shared objectives for the improvement of the nutritional characteristics of food products” (30 g/100 g and 1 g/100 g for sugar and salt, respectively) [14]. 

## 2. Materials and Methods 

### 2.1. Food Product Selection on Online Stores

Breakfast cereal-based products considered for the present work were selected from the major retailers present on the Italian market that have a home-shopping website (Auchan, Bennet, Carrefour, Conad, Coop Italia, Crai, Despar, Esselunga, Il Gigante, Iper, Pam Panorama, Selex, Sidis).

The online search for the information was conducted from July 2018 until December 2018. The selection of products was performed by considering the eligibility of the extraction of all the breakfast cereal items that were present in each online shop. 

The exclusion criteria for product selection were (i) non-prepacked foods, (ii) incomplete images of all the sides of the pack, (iii) unclear images of nutrition declaration or list of ingredients, (iv) products that were marked as ‘product currently unavailable’ on all the online stores selected during the whole data collection period.

### 2.2. Data Extraction

Data from the complete images of all the sides of the pack were collected for all included products. For each food item, the quali-quantitative and specifically regulated (mandatory) information was retrieved: company name, brand name, descriptive name, energy (kcal/100 g), total fat (g/100 g), saturates (g/100 g), carbohydrate (g/100 g), sugars (g/100 g), protein (g/100 g), and salt (g/100 g). Furthermore, other information, such as presence of NHC (presence or absence of at least one nutrition claim and presence or absence of at least one health claim) or GF declaration (presence or absence of gluten) was collected. 

The precision of the extracted data was double-checked by two researchers and inaccuracies were solved through secondary extractions with the help of a third researcher. 

A dataset was created with all the collected data and items were sub-grouped for specific comparisons by considering (i) descriptive name reported, (ii) presence/absence of GF declaration, and i(ii) presence/absence of NHC declaration. On the basis of the descriptive name, breakfast cereals were classified into six types: cereal bars, muesli, flakes, bran cereals, puffed cereals, and others (e.g., cereals with honey, cream-filled cereals). Definitions and examples of types and categories are provided in Table S1. 

### 2.3. Data Analysis

The statistical analysis was carried out using the Statistical Package for Social Sciences software (IBM SPSS Statistics, Version 25.0, IBM corp., Chicago, IL, USA) and performed at a significance level of *p* < 0.05. The normality of data distribution was rejected through the Kolmogorov–Smirnov test and variables were expressed as median (interquartile range). Data of energy and nutrient contents per 100 grams of products were analysed using the Kruskal–Wallis non-parametric one-way ANOVA for independent samples with multiple pairwise comparisons (for differences among types) and using the Mann–Whitney non-parametric test for two independent samples (for differences between GF declaration categories, nutrition claim categories, and health claim categories). In addition, a principal component analysis (PCA) with varimax rotation was performed for all items, considering energy and nutrient contents per 100 g of products to better describe the inter-product nutritional variability. 

Moreover, the sugar and salt content of the considered breakfast cereal products was compared with the mean amounts expected for 2017, as described in the “Shared objectives for the improvement of the nutritional characteristics of food products”: 30 g of sugar per 100 g and 1 g of salt per 100 g [14].

## 3. Results

### 3.1. Nutritional Composition of Breakfast Cereals

A total of 415 breakfast cereals were identified during the research conducted in the online stores. After removing the products on the basis of the exclusion criteria, a total of 371 different items were retrieved (Table 1). Thus, almost ~90% of the products sold in the considered retailers were retrieved. The inter-rater agreement in excluding of products was 98% and the remaining 2% of disagreements were successfully resolved by the third researcher.

Among these 371 items, products were mostly flakes (*n* = 129), followed by cereal bars (*n* = 78), muesli (*n* = 54), puffed cereals (*n* = 29), and lastly bran cereals (*n* = 14), whereas the remaining 67 items were classified as “others”, being very heterogeneous. Overall, the median energy content of breakfast cereals was 385 (372–417) kcal/100 g, but widely differed among types (*p* < 0.001). Indeed, energy content ranged from a median of 318 (301–344) kcal/100 g for bran cereals to 443 (381–463) kcal/100 g for muesli. Considering macronutrients, contents of total fat and saturates differed among the types (*p* < 0.001 for both), with the highest contents in muesli (15.8 (7.9–18.0) and 4.5 (1.7–6.0) g/100 g for total fat and saturates, respectively) and cereal bars (11.4 (7.9–20.0) and 4.2 (2.5–5.9) g/100 g for total fat and saturates, respectively). Total carbohydrate content differed among the types (*p* < 0.001), with the highest values for flakes (78.0 (67.0–81.0) g/100 g), puffed cereals (79.0 (75.9–84.0) g/100 g), and other breakfast cereals 73.0 (68.0–79.0) g/100 g). When sugars were taken into account, differences among the types were found (*p* < 0.001), with cereal bars and other breakfast cereals reporting the highest sugar contents: 27.0 (21.6–31.1) and 25.0 (20.0–29.7) g/100 g, respectively. Differences in protein content were observed among the cereal types (*p* < 0.001), with the bran cereals showing the highest content (14.9 (13.0–16.0) g/100 g) compared with the others. Salt content varied among the types (*p* < 0.001), with the flakes group having the highest content (0.8 (0.3–1.1) g/100 g), and muesli (0.3 (0.1–0.6) g/100 g) and puffed cereals (0.0 (0.0–0.7) g/100 g) the lowest content. 

No differences were identified when GF products were compared to the gluten counterparts.

Finally, breakfast cereals with at least nutritional claim resulted lower in total energy (*p* < 0.001) and sugars (*p* = 0.005) than their counterparts. Products carrying a health claim overall resulted as being lower in total carbohydrates (*p* = 0.005) and higher in protein content (*p* = 0.017) than the products without this declaration.

### 3.2. Inter-Product Variability of the Nutritional Composition of the Breakfast Cereals

**Figure 1:**
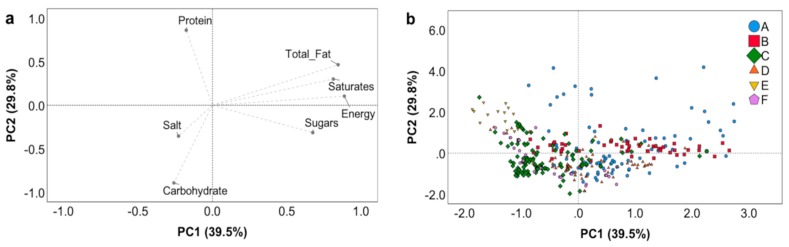
Principal component analysis (PCA) describing the inter-product variability based on the nutritional composition of products analysed (energy (kcal/100 g), total fat (g/100 g), saturates (g/100 g), carbohydrate (g/100 g), sugars (g/100 g), protein (g/100 g), and salt (g/100 g)). Loading plots of Principal Component (PC) 1 versus PC2 (**a**); score plots of the nutrition composition for each breakfast cereal product analysed from PC1 and PC2 (**b**). Legend: A, cereal bars; B, muesli; C, flakes; D, bran cereals; E, puffed cereals; F, others.

Differences in the nutritional profile of the breakfast cereal types were explained by two Principal Components (PCs), which described 69% of the total variability (Figure 1). Energy, total fat, saturates, and sugars were the nutritional variables with the highest contribution to PC1, which explained 40% of the total variability. PC2 described 30% of the inter-product variability, being loaded positively by protein and negatively by salt and carbohydrates (Figure 1A). A high inter-product variability was observed for all breakfast cereal types (Figure 1B), in particular for cereal bars, muesli, and puffed cereals. Products belonging to the bran cereal type were the ones that grouped better (negative scores for PC1 and positive ones for PC2), and they were characterized by a high amount of protein and low quantities of energy and other nutrients. Even if flake products were heterogeneous, the majority of them were described by a high content of carbohydrates and salt (negative scores for both PCs).

### 3.3. Comparison of the Sugar and Salt Contents of the Breakfast Cereals with the Italian Shared Objectives

**Figure 2:**
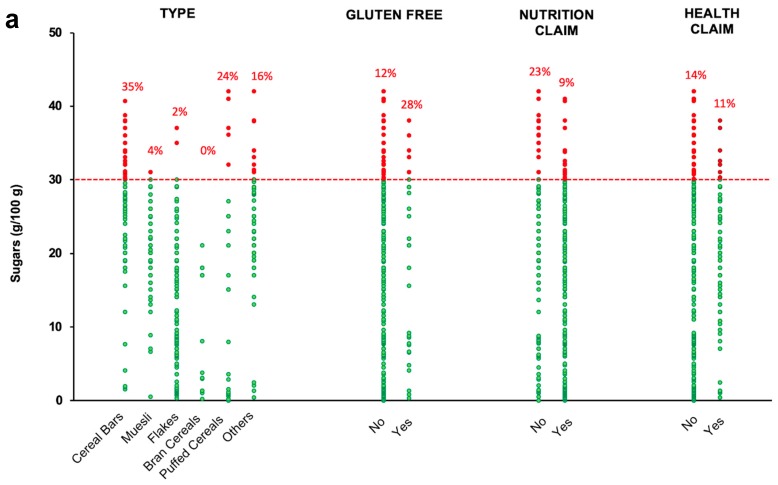

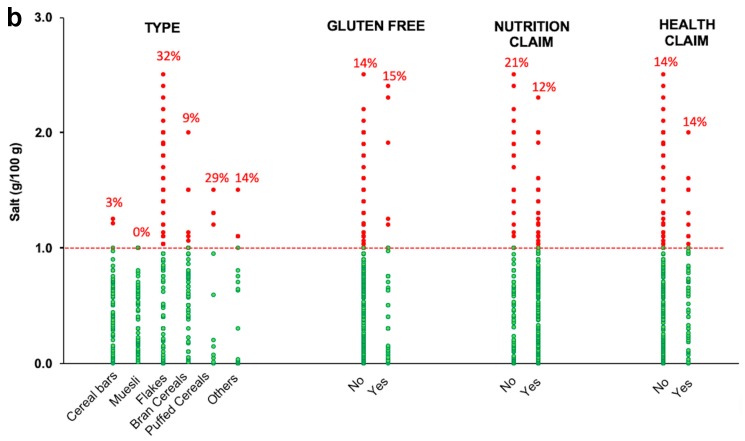
Sugar (**a**) and salt (**b**) content of the considered breakfast cereal products, classified per type, containing or not containing gluten, and carrying or not carrying nutrition and a health claim. Red dashed lines refer to the shared objectives for sugars (30 g/100 g, (**a**)) and salt (1 g/100 g, (**b**)) set by the Italian Ministry of Health [14]. Green and red dots represent the referring values of the product lower and higher, respectively, than the mean contents expected in the Italian Ministry of Health shared objectives. Percentage values on the top of each bar indicate the percentage number of the products with an amount of sugar (**a**) or salt (**b**) higher than the shared objectives.

In this survey, it emerged that most of the products matched the shared objectives for the improvement of the nutritional characteristics of products for both sugar (Figure 2a) and salt contents (Figure 2b) [14]. However, the percentage of products having a sugar content lower than 30 g/100 g ranged from 65% of the cereal bars to 98% of flakes and 100% of bran cereals. Although the number of GF and gluten-containing breakfast cereals was different (33 vs. 338, respectively), 72% and 88% of the products, respectively, had sugars lower than 30 g/100 g. Considering products with >30 g sugar/100 g, all GF products were cereal bars, whereas, among non GF products, ~50% were cereal bars and ~17% were puffed cereals (data not shown). 

Similarly, products carrying or not carrying NHC had more than 75% of the products matching the objective of <30 g sugars/100 g, reaching 91% for products carrying at least a nutrition claim. Once again, most of the products with >30 g sugar/100 g were cereals bars (e.g., 16 out of 23 items with nutrition claims and 5 out of 7 items with health claims, data not shown).

Similarly to sugars, for all the breakfast cereal types, most of the products have a salt content lower than the objective of 1 g/100 g, ranging from 68% of the flakes to 100% of muesli products (Figure 2b). Both GF and gluten-containing breakfast cereals included more than 80% of the products below the objective of 1 g salt/100 g. It is worth noting that 4 out of 5 GF items and 33 out of 48 non-GF items with salt >1 g/100 g were flakes (data not shown).

Finally, 88% of products carrying nutrition claims, 79% of products without them, and 86% of products both with and without health claims were below the objective value for salt. Again, products above the threshold for salt were mostly flakes (e.g., 19/30 and 4/9 among items with nutrition and health claims, respectively) (data not shown).

## 4. Discussion

The inclusion in the breakfast of cereal-based products has been demonstrated to be a valid choice for increasing the nutritional quality of one’s diet [22,23]. Despite their health effects, breakfast cereals are a heterogeneous category of food products and different international surveys report a great variability in their nutritional composition, mostly for sugar and salt content [12,24]. The present survey was aimed at giving an overview of the nutritional quality of the breakfast cereals sold on the Italian market, with particular focus on the differences among types, as well as among products with or without NHC and gluten declarations. Results evidenced a great variability of the nutritional values among the different types of products. Muesli products have shown the highest median energy as well as the highest total fat and saturate median contents. These values are in line with the ones of the items sold in French [25,26] and New Zealand [13] markets, as well as with the data shown in a comparative survey among muesli sold in Austria, France, and Romania [27]. Conversely, bran cereals sold in Italy have the highest content of protein but the lowest amount of energy and sugars compared to the other five Italian breakfast types. These median data of energy are slightly lower than those previously reported for the bran products, where total energy content of brans was on average around 348 kcal/100 g [13]. Intriguingly, the mean sugar content for these products sold in the New Zealand market was notably higher than the one found in the Italian products (22.5 g/100 g vs. 3.4 g/100 g), but it is worth noting that we found a greater inter-product variability, with a maximum value of 21 g/100 g. Similar results in terms of variability of sugar and total fat contents have been found for flakes, which are characterized by a high carbohydrate and salt content. These findings are in line with the ones found in a recent comparative survey among three European countries for oat flakes [27].

Taking into account the great difference in terms of numbers of items sold on the Italian market, there are no significant differences among the energy, macronutrient, and salt contents of breakfast cereals containing or not containing gluten. Previous surveys investigating the nutritional profiles of breakfast cereals with or without gluten often found opposite results. For instance, our data are partially contrasting with the ones of a U.K. survey that found lower sugar and salt contents in GF breakfast cereals compared to their gluten counterpart [28]. A lower salt content in GF cereal bars compared to the regular ones was also found in an Australian survey, whereas energy and sugars were lower in the products containing gluten [29]. Again, it is worth underlining that the present data are not sufficient for a thorough evaluation of the nutritional quality of GF breakfast cereals, as some other aspects such as the ingredient list and micronutrient contents should be considered [30]. 

Regarding the presence of NHC, a strict regulation on front/back-of-pack label information could be useful to deliver the correct nutritional and health information, as also suggested by García et al. in 2019 [31]. This is particularly important considering that NHC may play a role on the customer’s intention to buy [32]. The EU Project “Food Labelling to Advance Better Education for Life” concluded that, even before the release of the European Regulation (EU) no. 1169/2011 concerning the mandatory information on foods [15], that breakfast cereals were the products with the highest penetration of nutrition information within the 27 EU countries [19]. In fact, by considering 6275 breakfast cereal products, nutritional information (i.e., NHC, labelling schemes such as traffic lights, guideline daily amounts other than nutrition declaration) were present on the back-of-pack of 94% of the items and on the front-of-pack of 70% of the items [19]. In this scenario, a recent Canadian study showed that, despite breakfast cereals being marked as “healthy food choices” and often boasting NHC on the front-of-pack, customers mainly find unhealthy products promoted in Canadian supermarkets [33]. Our findings concerning products depicting NHC confirm that there are no deep differences in terms of nutritional profile compared to breakfast cereals with no NHC. Breakfast cereals carrying nutrition claims showed only a 3% lower median energy content compared to the products not claiming nutrition, mainly due to a lower sugar content. Items boasting health claims were slightly, but significantly, lower in total carbohydrates and higher in protein contents than those not presenting a claim. However, it is worth noting that the number of items carrying a health claim was five-fold lower compared to the number of items without, which represents one of the main limitations of this study. These findings support the evidence of several surveys in the United Kingdom, USA, and New Zealand, which concretely demonstrated that products, in particular breakfast cereals, boasting nutrition and/or health claims do not necessarily have an overall better nutritional profile [31,34,35,36]. The absence of clear and marked differences among products with and without NHC may be because food items with NHC do not have to comply with any nutrient profile. In this scenario, it is worth noting that the Article 4 of the EU Regulation 1924/2006 stated that the European Commission should have established by 19 January 2009 specific nutrient profiles that foods or certain groups of foods should have respected in order to bear nutrition and health claims [16]. However, in 2016, the European parliament voted to scrap nutrient profile. As a consequence, manufacturers currently do not have to follow specific nutrient profile regulations to formulate products bearing NHC.

Despite this, food companies should formulate food products with the highest nutritional quality possible. The World Cancer Research Fund International considered that the nutritional reformulation of products is one of the main tools necessary to drastically reduce obesity and non-communicable diseases [37]. As already mentioned, the Italian Ministry of Health, in collaboration with food companies, initiated a process for the improvement of the nutritional characteristics of food products [14]. It is worth underlying that the shared objectives with manufacturers are just a first and not resolutive step for the production of nutritionally balanced breakfast cereal products. For example, by considering an expected mean content of 30 g sugars/100 g of breakfast cereals [14] and a reference serving size of 30 g, one portion of breakfast cereals provides on average ~9 g sugars. Considering that no more than 15% of the daily energy intake should come from sugars [38], ~9 g sugars from breakfast cereals corresponds to 12% of the daily amounts of sugar for a 2000 kcal-diet.

Similarly, 1 g salt per 100 g product means ~300 mg salt in a 30 g cereal serving, which is roughly 6% of the daily salt intake, considering 5 g per day as the suggested dietary target [39]. Again, these values suggest that further steps are needed in order to reach lower values for sugar and salt contents in breakfast cereal products.

In the present survey, it has been found that, despite the high variability in terms of the nutritional profile of the six breakfast cereal types, and regardless of whether they contained gluten or carried an NHC, most of the products were below the suggested targets for sugars and salt. However, at least 25% and 13% of the 371 considered products have a salt and sugar content higher than the objectives, respectively. This information supports that further reformulation is desirable for offering the consumers products with an improved nutrient profile.

## 5. Conclusions

The present work clearly highlighted the high variability in the nutritional profile among different types of breakfast cereals sold on the Italian market. On the whole, results showed that the boasting of NHC or declarations on gluten on the labels did not necessarily indicate a better nutritional quality of the product. Most importantly, these results support the need of an informative labelling on food products to help consumers to make informed food choices. Moreover, the results support the importance of a nutritional education towards a better understanding of food labels as a key point to help the consumer in making healthy food choices. In addition, one of the aspects of the present work was the evaluation of the nutritional profile of the products, mainly focusing on sugars and salt. In Italy, an initial step for the reformulation of different product categories—among which are breakfast cereals—has been jointly enacted by the Italian Ministry of Health and manufacturers. However, for a complete, accurate, and science-based reformulation process, it would be worthy to set-up a durable working group involving all the stakeholders, that is, industries, institutions, and scientific societies.

However, because this study considered only breakfast cereals, future surveys focused on other food groups are needed to draw a more accurate nutritional profile of food products currently on the Italian market. Moreover, in the present study, other retail channels, such as discounts, were not considered and would be worthy of future investigation. This would further increase the number of items in the study to better understand the potential role of food declarations as markers of the overall quality of food products. Lastly, it is advisable to replicate this research study on a regular basis in order to investigate the impact of reformulation on the nutritional quality of breakfast cereals.

## Figures and Tables

**Table 1:** Energy, macronutrients, and salt across breakfast cereal categories.

		Number of Items	Energy kcal/100 g	Total Fat g/100 g	Saturates g/100 g	Total Carbohydrates g/100 g	Sugars g/100 g	Protein g/100 g	Salt g/100 g
**Category**	Breakfast cereals	371	385 (372–417)	5.5 (2.5–13.5)	1.5 (0.5–3.8)	69.0 (61.0–79.0)	20.0 (8.6–27.0)	8.3 (7.0–10.8)	0.5 (0.2–0.8)
**Type**	Cereal bars	78	400 (383–448) ^a^	11.4 (7.9–20.0) ^a^	4.2 (2.5–5.9) ^a^	64.2 (49.0–69.7) ^b^	27.0 (21.6–31.1) ^a^	7.8 (6.1–11.5) ^b^	0.5 (0.3–0.7) ^b,c^
	Muesli	54	443 (381–463) ^a^	15.8 (7.9–18.0) ^a^	4.5 (1.7–6.0) ^a^	62.0 (60.0–65.0) ^b,c^	21.0 (18.0–25.0) ^b,c^	8.9 (8.0–9.5) ^b^	0.3 (0.1–0.6) ^c^
	Flakes	129	377 (371–385) ^c^	2.0 (1.2–5.6) ^c^	0.5 (0.3–1.3) ^c^	78.0 (67.0–81.0) ^a^	10.8 (6.0–17.7) ^d^	8.4 (7.4–11.0) ^b^	0.8 (0.3–1.1) ^a^
	Bran cereals	14	318 (301–344) ^d^	4.3 (3.9–7.3) ^b,c^	0.9 (0.7–1.2) ^b,c^	40.2 (34.0–48.0) ^c^	3.4 (1.3–17.0) ^d^	14.9 (13.0–16.0) ^a^	0.2 (0.0–1.2) ^a,b^
	Puffed cereals	29	381 (368–397) ^b,c^	2.9 (1.9–4.0) ^c^	0.6 (0.5–1.0) ^c^	79.0 (75.9–84.0) ^a^	15.0 (0.9–27.0) ^c,d^	7.0 (6.9–9.9) ^b^	0.0 (0.0–0.7) ^c^
	Others	67	392 (382–437) ^a,b^	4.4 (2.9–14.0) ^b^	1.6 (1.0–3.1) ^b^	73.0 (68.0–79.0) ^a^	25.0 (20.0–29.7) ^a,b^	8.0 (6.9–9.0) ^b^	0.6 (0.3–0.8) ^a,b^
**Gluten free**	No	338	385 (372–416)	5.5 (2.5–12.2)	1.4 (0.5–3.8)	69.0 (62.0–79.0)	20.0 (9.0–26.5)	8.4 (7.0–10.0)	0.5 (0.2–0.8)
	Yes	33	390 (375–448)	5.9 (2.5–17.0)	1.9 (0.6–3.8)	71.0 (51.0–81.0)	21.0 (7.7–30.0)	8.0 (7.1–11.0)	0.5 (0.1–0.8)
**Nutrition claim**	No	112	393 (378–449) ^a^	5.3 (2.6–16.0)	1.8 (0.6–4.3)	72.0 (62.0–80.4)	22.0 (8.2–30.0) ^a^	8.0 (7.0–10.0)	0.5 (0.1–1.0)
	Yes	259	382 (371–407) ^b^	5.9 (2.5–11.2)	1.3 (0.5–3.5)	68.0 (60.3–78.7)	19.0 (9.0–25.0) ^b^	8.4 (7.1–11.0)	0.5 (0.2–0.8)
**Health claim**	No	306	385 (372–422)	5.3 (2.3–14.0)	1.4 (0.5–3.8)	70.0 (62.0–79.5) ^a^	20.0 (7.9–27.0)	8.1 (7.0–10.0) ^b^	0.5 (0.2–0.8)
	Yes	65	383 (373–410)	6.9 (2.6–10.3)	1.6 (0.7–3.4)	65.0 (56.0–74.8) ^b^	20.0 (14.0–27.0)	9.0 (7.4–12.5) ^a^	0.7 (0.3–0.8)

Values are expressed as median (25th–75th percentile). For each category, different lowercase letters in the same column indicate significant differences among type (Kruskal–Wallis non-parametric one-way ANOVA for independent samples with multiple pairwise comparisons) or between groups (gluten free, nutrition claim, health claim; Mann–Whitney non-parametric test for two independent samples), *p* < 0.05.

## Supplementary Materials

The following are available online at https://www.mdpi.com/2072-6643/11/11/2827/s1: Table S1: Definitions and examples of the categorization of the breakfast cereals.

## SINU Young Working Group

Marika Dello Russo	Institute of Food Sciences, National Research Council, Avellino, Italy
Stefania Moccia	Institute of Food Sciences, National Research Council, Avellino, Italy
Daniele Nucci	Veneto Institute of Oncology IOV-IRCCS, Padova, Italy
Gaetana Paolella	Department of Chemistry and Biology A. Zambelli, University of Salerno, Fisciano, Italy
Veronica Pignone	Department of Epidemiology and Prevention, IRCCS Neuromed, Pozzilli, Italy
Emilia Ruggiero	Department of Epidemiology and Prevention, IRCCS Neuromed, Pozzilli, Italy
Carmela Spagnuolo	Institute of Food Sciences, National Research Council, Avellino, Italy
