# Supplementary material for: Evaluation of the Nutritional Quality of Breakfast Cereals Sold on the Italian Market: The Food Labelling of Italian Products (FLIP) Study
Source: Nutrients. 2019 Nov 19;11(11):2827. doi: 10.3390/nu11112827 (PMC6893738; doi:10.3390/nu11112827)
Supplement: Supplementary file 1 [file nutrients-11-02827-s001.pdf]

Supplementary Table 1. Definition and examples of the categorization of the breakfast cereals.

		DEFINITION AND EXAMPLES
**Type**	Bars	Cereals made with one or more cereal grains (e.g. oat, rice, wheat) and other ingredients (e.g. dried fruits, nuts, chocolate) and shaped as bars
	Muesli	Cereals typically made with rolled oat and other cereal grains (e.g. corn, rice, wheat) and other ingredients (e.g. dried fruits, nuts, chocolate)
	Flakes	Cereals produced with different rolled out and toasted cereals (e.g. corn flakes, wheat flakes)
	Bran cereals	Bran or bran-based cereals obtained by different grains (e.g. oat, wheat) and sometimes available in different shapes (e.g. sticks)
	Puffed cereals	Cereals obtained by an expansion process of different cereal grains (e.g. corn, wheat) and sometimes added with other ingredients (mostly cocoa)
	Others	All the other items not compliant with the above definitions (e.g. cream-filled cereals, cereals with honey)
**Gluten free**	No	Gluten-containing items (e.g. wheat-based breakfast cereals)
	Yes	Cereals made with cereal grains naturally free of gluten (e.g. rice-based cereals)
**Nutrition claim**	No	Products not carrying nutrition claim as defined by the European Regulation (CE) No. 1924/2006
	Yes	Products carrying at least one nutrition claim as defined by the European Regulation (CE) No. 1924/2006 (e.g. "rich in fiber", "source of vitamin B6")
**Health Claim**	No	Products not carrying health claim as defined by the European Regulation (CE) No. 1924/2006
	Yes	Products carrying at least one nutrition claim as defined by the European Regulation (CE) No. 1924/2006 (e.g. "protein contributes to the maintenance of muscle mass")
